# The Effects of Prolonged Water-Only Fasting and Refeeding on Markers of Cardiometabolic Risk

**DOI:** 10.3390/nu14061183

**Published:** 2022-03-11

**Authors:** Eugene Scharf, Evelyn Zeiler, Mackson Ncube, Patricia Kolbe, Su-Yeon Hwang, Alan Goldhamer, Toshia R. Myers

**Affiliations:** 1 Department of Neurology, Mayo Clinic, Rochester, MN 55902, USA; 2 Department of Research, TrueNorth Health Foundation, Santa Rosa, CA 95404, USA; drzeiler@truenorthhealthfoundation.org (E.Z.); macksonncube@gmail.com (M.N.); patricia@truenorthhealthfoundation.org (P.K.); suyeon_hwang@my.uri.edu (S.-Y.H.); dracg@truenorthhealth.com (A.G.); drmyers@truenorthhealth.org (T.R.M.); 3 TrueNorth Health Center, Santa Rosa, CA 95404, USA

**Keywords:** prolonged fasting, cardiometabolic health, insulin resistance, hypertension, hyperlipidemia, plant diet

## Abstract

(1) Background: Cardiometabolic disease, including insulin resistance, hyperlipidemia, and hypertension, are major contributors to adverse health outcomes. Fasting has gained interest as a nonpharmacological therapeutic adjunct for these disorders. (2) Methods: We conducted a prospective, single-center study on the effects of prolonged water-only fasting followed by an exclusively whole-plant-food refeeding diet on accepted measures of cardiovascular risk and metabolic health. Participants were recruited from patients who had voluntarily elected to complete a water-only fast in order to improve their overall health according to an established protocol at an independent, residential medical center. Median fasting and refeed lengths were 17 and 8 days, respectively. The primary endpoint was to describe the mean glucose tolerance as indicated by Homeostatic Model Assessment of Insulin Resistance (HOMA-IR) scores at baseline, end-of-fast (EOF), and end-of-refeed (EOR) visits. Secondary endpoints were to describe the mean weight, body mass index (BMI), abdominal circumference (AC), systolic blood pressure (SBP), diastolic blood pressure (DBP), lipid panel, and high-sensitivity C-reactive protein (hsCRP) at the same time points. (3) Results: The study enrolled 48 overweight/obese non-diabetic participants, of which 26 completed the full study protocol. At the EOF visit, the median SBP, AC, low-density lipoprotein (LDL), and hsCRP were decreased and triglycerides (TG) and HOMA-IR scores were increased. Conclusion: Prolonged water-only fasting and whole-plant-food refeeding holds potential as a clinical therapy for cardiometabolic disease but increased TG and HOMA-IR values after refeeding necessitate further inquiry.

## 1. Introduction

Cardiovascular and metabolic diseases are among the leading causes of death and disability globally [1]. Although pathologically distinct, these diseases share insulin resistance as a key driver during early pathogenesis [2]. Obesity is a primary cause of insulin resistance [3], with adipocyte dysfunction affecting adipokine secretion, lipid metabolism, glucose uptake, insulin sensitivity, and transcriptional regulation in adipocytes, which may impact systemic insulin response and contribute to insulin resistance in liver and muscle cells as well [4]. The World Obesity Federation has estimated that global obesity rates have nearly tripled in the past 30 years, and it is estimated that obesity will affect more than 1 billion adults by 2025 (https://www.worldobesity.org, accessed on 26 February 2022) even though the disease is preventable with diet and lifestyle interventions [5]. Treatment strategies that efficiently reduce adipose tissue while improving insulin response are necessary.

Fasting is the partial or total abstention from food and/or liquid for periods ranging anywhere from a few hours each day up to several weeks each year. Intermittent fasting and prolonged very-low-calorie fasting methods have emerged as potential treatments for obesity and cardiometabolic dysfunction [5]. Reported health benefits may be because even brief periods of fasting (e.g., overnight) reduce glucose levels sufficiently to increase the production of ketone bodies [5,6]. Ketone bodies are purported to regulate processes ranging from histone deacetylase inhibition [7] to mitohormesis [8] that may affect metabolic health. During prolonged fasting periods, ketone levels plateau after approximately 2 weeks and can theoretically continue meeting the bulk of total energy requirements for as many as 90 days [9].

Therapeutic water-only fasting is prolonged zero-calorie fasting practiced under medical supervision that requires a controlled refeeding diet to avoid complications from refeeding syndrome [10]. We previously reported that this fasting method, including the gradual re-introduction of exclusively whole-plant foods without added salt, oil, or sugar, is tolerable with a low risk of causing a serious adverse event [10]. Preliminary research suggests that prolonged water-only fasting may improve markers of cardiometabolic health, such as weight [11], blood pressure [12,13], leptin [14], glucose, and insulin [15,16,17]. However, data on the effects of fasting on cardiometabolic markers, including insulin sensitivity, during the post-fast refeeding period are sparse [18]. To this end, we conducted a single-arm, open-label, observational study on the effects of at least 10 days of water-only fasting, followed by at least 5 days of refeeding with an exclusively whole-plant-food diet on the homeostatic model of the insulin resistance (HOMA-IR) index, which is calculated from fasting glucose and insulin and used to approximate insulin resistance in overweight/obese non-diabetic participants.

## 2. Materials and Methods

Forty-eight overweight and obese participants were recruited from patients voluntarily undergoing elective, medically supervised, water-only fasting for various medical reasons (Supplemental Table S1) at a residential fasting center in Santa Rosa, CA, USA. Consenting participants of any sex, between 40 and 70 years old, with a fasting glucose < 6.99 mmol/L and/or hemoglobin A1c < 7% and a body mass index (BMI) > 25 kg/m^2^ who were approved by a non-research clinician for water-only fasting for at least 10 consecutive days followed by refeed of at least 5 days were eligible for inclusion. Exclusion criteria included active malignancy; active inflammatory disorder, including classic autoimmune connective tissue disorders; multiple sclerosis; inflammatory bowel disorders; and stroke or heart attack within the last 90 days.

The medically supervised, water-only fasting and exclusively whole-plant-food refeeding protocol took place at a residential, integrative medical facility and was administered by non-research medical personnel according to previous methods (10 and Supplemental Methods). Briefly, patients were instructed to consume a pre-fast elimination diet consisting of only cooked and raw fruits and vegetables for at least 2 days prior to fasting. During the water-only fast, patients consumed a minimum of 40 ounces of steam distilled water daily and limited physical exertion. Vital signs were examined by medical personnel twice daily, and labs were monitored weekly or as directed by the attending physician. Water-only fasts were broken with the gradual re-introduction of exclusively whole-plant foods free of added salt, oil, and sugar. The refeed plan consisted of five phases: phase 1, fruit and vegetable juices and/or vegetable broths; phase 2, raw fruits and vegetables and/or steamed squash; phase 3, raw fruits and raw and/or steamed vegetables; phase 4, raw fruits and raw and/or steamed vegetables and grains; and phase 5, unrestricted whole-plant foods free of added salt, oil, and sugar. Each phase lasted 1 day per 7–10 days of water-only fasting for a total of at least half of the fasting length. For the purposes of this study, fixed-minimum fasting and refeeding lengths were set at 10 and 5 days, respectively.

Participants reported for study visits at baseline, every 7th day of fasting and refeeding, end of fast (EOF), and end of refeed (EOR). Treatment status (i.e., prefeeding, fasting, or refeeding), resting systolic blood pressure (SBP), resting diastolic blood pressure (DBP), weight, abdominal circumference (AC), and 28.5 mL of blood were obtained at each study visit. At baseline, demographic information and height were also obtained. Sera were sent to LabCorp for fasting glucose, insulin, high-sensitivity C-reactive protein (hsCRP), and lipid panel analyses.

Height (cm) was measured at the baseline visit with a wall-mounted stadiometer from Doran Scales Inc. Participants were not wearing shoes. The weight, the abdominal circumference, and the resting blood pressure were obtained at each visit. The weight (kg) was measured using a digital body scale from Tanita (BWB 800A Class III). The participants wore a single layer of clothing but were not wearing shoes, jackets, extra sweaters, etc., and they were instructed to wear similarly weighted clothing during future visits. The BMI (kg/m^2^) was calculated using the formula weight (kg) ÷ height (m^2^).

The abdominal circumference was measured on bare skin at the minimal waistline with a tension-sensitive, non-elastic tape (Gullick II, Model 67019). The tape was placed horizontally and parallel to the floor around the abdomen and the measurement was read on the right side of the body, in the midaxillary line, and at the end of a normal expiration.

The resting blood pressure was measured with a Connex ProBP Digital Blood Pressure Device 3400 from Welch Allyn with the participant resting and in a sitting position. Different arm cuffs were used according to the arm size: small adult 10 for arms measuring 20–26 cm (light-blue cuff), adult 11 for arms measuring 25–34 cm (dark-blue cuff), and large adult 12 for arms measuring 32–43 cm (red cuff).

Blood was drawn in the morning, before caloric food or liquid consumption, and in a sitting position by a certified phlebotomist. The participants were instructed to drink 1–2 cups of water before the blood draw into BD Vacutainer tubes (Lavender top, 16 × 100, 10 mL, K2EDTA; Red top, 16 × 100, 10 mL, silica; Tiger top, 16 × 100, 8.5 mL, silica, polymer gel). The lavender top vacutainer tube was placed on ice immediately and centrifuged at 1500× *g* for 10 min at 4 °C. The plasma was separated and frozen at −80 °C. The red top and tiger top tubes were incubated at room temperature for 30 min before centrifugation at 1500× *g* for 10 min at 4 °C. The serum was separated and was either frozen at −80 °C or refrigerated at 4 °C and sent to LabCorp for analysis. LabCorp reports that glucose was measured by enzymatic assay using hexokinase and photometrically on Roche/Hitachi cobas c systems. Insulin levels were measured by electrochemiluminescence immunoassay on cobas e immunoassay analyzers from Roche Diagnostics. Total cholesterol (TC), high-density lipoprotein (HDL), and triglycerides (TG) were measured by enzymatic colorimetric assay on Roche/Hitachi cobas c systems. Low-density lipoprotein (LDL) and very-low-density lipoprotein (VLDL) were calculated using the Friedewald equation and estimated as 20% of total triglycerides, respectively. hsCRP was measured by a high-sensitivity-particle-enhanced immunoturbidimetric assay on Roche/Hitachi cobas c systems.

The study primary endpoint, HOMA-IR, was calculated using glucose and insulin values (fasting insulin (microU/L) × fasting glucose (nmol/L)/22.5) according to prior methods [19]. The prespecified primary analysis was pooled unadjusted change in HOMA-IR across three paired time points, baseline, end of fast, and end of refeed, assessed by repeated measures ANOVA with a paired *t*-test post hoc analysis. HOMA-IR and other cardiometabolic markers were assessed across baseline, EOF, and EOR by the Friedman test with a Wilcoxon signed-rank test post hoc. The Friedman test was used since many cardiometabolic markers contained outliers, identified using boxplot methods [20].

Secondary analyses were performed by the non-parametric univariate Siegel repeated medians model [21] to investigate the relationship between changes in clinical characteristics with length of fast, length of refeed, or total length of fast and refeed (referred to as total length in data tables) as well as the relationship between change in total cholesterol and change (from baseline to EOR) in LDL, HDL, VLDL, and triglycerides. Changes in clinical characteristics were estimated between baseline and EOR for weight, BMI, abdominal circumference, blood pressure, circulating lipid levels, hsCRP, glucose, insulin, and HOMA-IR; in addition, changes in HOMA-IR were estimated between baseline and EOF. Siegel repeated medians were used due to outliers, identified using residuals versus leverage plots [22] and normality violations identified by the Shapiro–Wilk normality test. Due to extreme outliers and normality violations, HOMA-IR scores were log transformed prior to inclusion in repeated measures ANOVA and linear regression models. All hypothesis tests in this report were two-sided with α-level 0.05. The Bonferroni correction was used for multiple comparisons, but the correction was only applied to post hoc analyses (i.e., only the Wilcoxon signed-rank test and the paired *t*-test). Clinical significance was noted if there was precedence.

Most of the primary and secondary models presented in this study are non-parametric. When the assumptions of a parametric model are satisfied, then the parametric model has the greatest ability to detect true differences in the given sample. However, in situations with small violations, it is often not clear if parametric or non-parametric models perform better. Given the small sample size and the observed parametric violations, this study contains sensitivity models that can help understand inferences produced by a range of models. Sensitivity analyses were conducted to understand how the models described above agree with inferences from their common alternatives [23]. The alternative models include repeated measures ANOVA for all models using the Friedman test (Supplemental Material Table S2), change score regression (without and with outliers; Supplemental Material Tables S3 and S4), and baseline-adjusted regression (without and with outliers; Supplemental Material Tables S5 and S6) for all models using Siegel repeated medians [24]. The sensitivity analyses check for two cases of divergence between the main analysis and the sensitivity analysis. One case is where one result is statistically significant but the other is not; in Supplemental Material, these cases are flagged with the symbol λ. The second case is where the directions of statistically significant coefficients diverge; these cases are flagged by the symbol ζ in Supplemental Material. See Diagnostic Supplement for complete results from the diagnostic analysis. Statistical analyses were performed using R version 4.1.1 [25] and JMP^®^, Version <14.1.0> (SAS Institute Inc., Cary, NC, USA, 1989–2019). The R scripts and output used in this study are available upon request.

## 3. Results

### 3.1. Study Population and Enrollment

**Figure 1:**
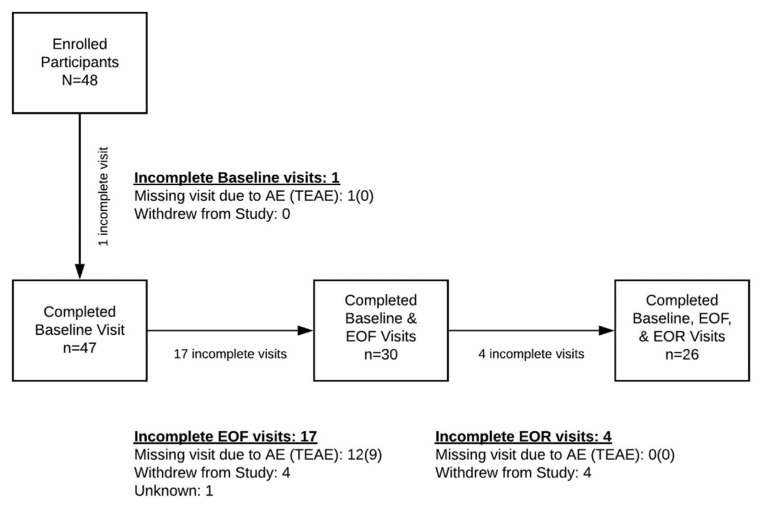
Enrollment diagram. EOF, end of fast; EOR, end of refeed; AE, adverse event; TEAE, treatment emergent adverse event. See Results Section 3.1 for a list of AEs.

Fifty-four percent (26/48) of the participants completed the full study protocol (Figure 1). Of the 22 participants who did not complete the study requirements, 9 withdrew for reasons unrelated to fasting; 1 withdrew for unknown reasons; and 13 were withdrawn because they were unable to complete the minimally required fasting length due to adverse events, 9 of which were treatment emergent adverse events, including headache, feeling unwell, acid reflux, fatigue, anxiety, cramping, panic attack, vomiting, and heart palpitations, and 4 of which were probably not due to treatment, including the inability to stop medication use due to vomiting, migraine, family stress, and dental infection. No severe or serious adverse events were reported during fasting or refeeding. Of the 26 participants, 20 were female and the median (range) age was 57 (42–68) years old. The median (range) fasting and refeed lengths were 17 (10–30) and 8 (5–25) days, respectively. Baseline characteristics of enrolled and withdrawn participants are shown in Supplemental Table S1; there were no clinically meaningful differences in sex, age, BMI, or resting BPs between the two groups.

### 3.2. Weight, BMI, and Abdominal Circumference

**Figure 2:**
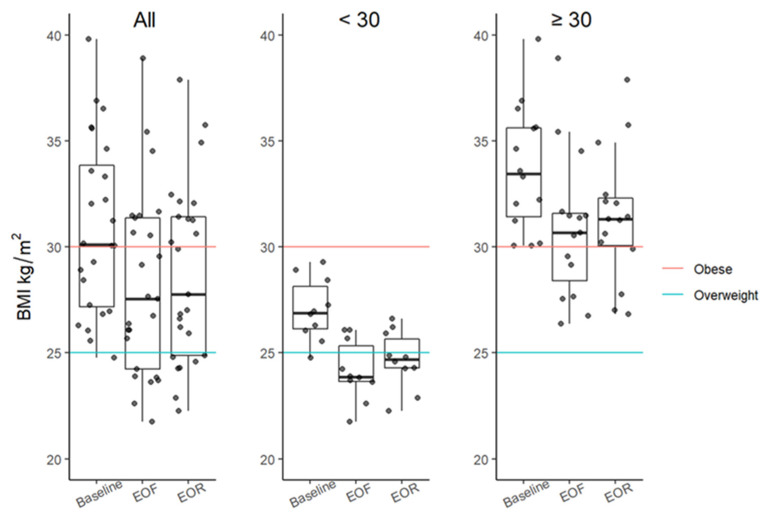
BMI boxplots at baseline, EOF, and EOR. Data further categorized based on obesity threshold (≥30 kg/m^2^; red line). A BMI between 25 kg/m^2^ (blue line) and 29 kg/m^2^ is overweight. Boxplots represent the minimum value, first (lower) and third (upper) quartiles, the median, and the maximum value. EOF, end of fast; EOR, end of refeed; BMI, body mass index; kg, kilogram; m, meter.

There were clinically meaningful reductions in weight, BMI, and abdominal circumference at EOF that were sustained at EOR. Changes in weight after fasting and refeeding were significant for each time point with a median of differences (MOD) of −9 kg (*p* < 0.0001), −7.3 kg, (*p* < 0.0001), and of 1.3 kg (*p* = 0.0006) from baseline to EOF, baseline to EOR, and EOF to EOR, respectively (Table 1). It follows that differences in the BMI were also significant for each time point, with MODs of −3.2 kg/m^2^ (*p* < 0.0001), −2.5 kg/m^2^ (*p* < 0.0001), and 0.5 kg/m^2^ (*p* = 0.0001) from baseline to EOF, baseline to EOR, and EOF to EOR, respectively (Figure 2; Table 1). Out of 9 participants who were overweight at baseline, 6 were normal weight at EOR; moreover, out of 16 participants who were obese at baseline, 4 were overweight at EOR. A 1-day increase in fasting duration was associated with a 0.39 kg reduction in weight (*p* = 0.0002) and a 0.16 kg/m^2^ reduction in BMI (*p* < 0.0001). A 1-day increase in refeed duration was associated with a 0.91 kg reduction in weight (*p* < 0.0001) and a 0.38 kg/m^2^ reduction in BMI (*p* < 0.0001). A 1-day increase in total intervention duration (i.e., fasting duration plus refeed duration) was associated with a 0.29 kg reduction in weight (*p* < 0.0001) and a 0.12 kg/m^2^ reduction in BMI (*p* < 0.0001).

The median AC decreased from 97.6 cm at baseline to 90.7 cm and 90.8 cm at EOF and EOR, respectively. These differences were significant with MODs of −7.1 cm (*p* < 0.0001), −5.7 cm (*p* < 0.0001), and 1.7 cm (*p* = 0.0054) from baseline to EOF and baseline to EOR, respectively (Table 1 and Supplemental Table S2). A 1-day increase in fasting duration, refeed duration, and total duration was, respectively, associated with a 0.36, 0.65, and 0.22 cm reduction in the AC from baseline to EOR (*p* < 0.0001 for each model; Supplemental Table S7).

### 3.3. Resting Blood Pressure

**Figure 3:**
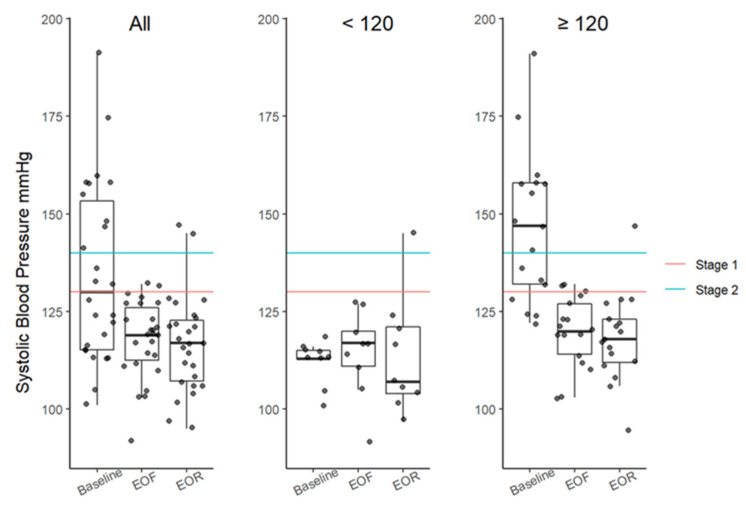
SBP boxplots at baseline, EOF, and EOR. Data categorized based on the risk threshold (≥120 mmHg). SBP between 130 mmHg (red line) and 139 mmHg is considered stage 1 hypertension. SBP ≥140 mmHg (blue line) is considered stage 2 hypertension. Boxplots represent the minimum value, first (lower) and third (upper) quartiles, the median, and the maximum value. EOF, end of fast; EOR, end of refeed; SBP, systolic blood pressure; mmHg, millimeters of mercury.

**Figure 4:**
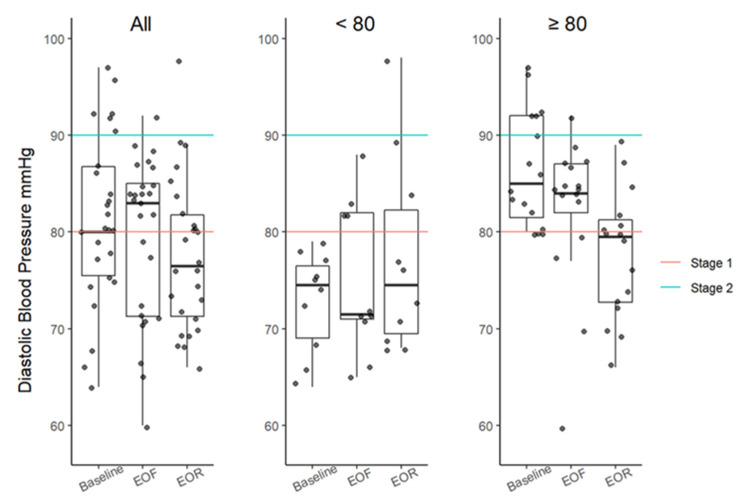
DBP boxplots at baseline, EOF, and EOR. Data further categorized based on high threshold (≥80 mmHg; red line). DBP between 80 and 89 mmHg is considered stage 1 hypertension. SBP ≥90 mmHg (blue line) is considered stage 2 hypertension. Boxplots represent the minimum value, first (lower) and third (upper) quartiles, the median, and the maximum value. EOF, end of fast; EOR, end of refeed; DBP, diastolic blood pressure; mmHg, millimeters of mercury.

There were clinically meaningful reductions in median systolic and diastolic blood pressures (Figure 3 and Figure 4; Table 1). The differences in the SBP from baseline to EOF and EOR were significant with MODs of −14 mmHg (*p* = 0.0054) and −13 mmHg (*p* = 0.0012; Table 1), respectively. Changes in the DBP were found to be significant using the Friedman test (Fr = 7; *p* = 0.0368; Table 1) but RMA sensitivity (*p* = 0.1280; Supplemental Table S2) and Wilcoxon signed-rank test did not yield significant results for any time point (Table 1).

### 3.4. Serum Lipids

**Figure 5:**
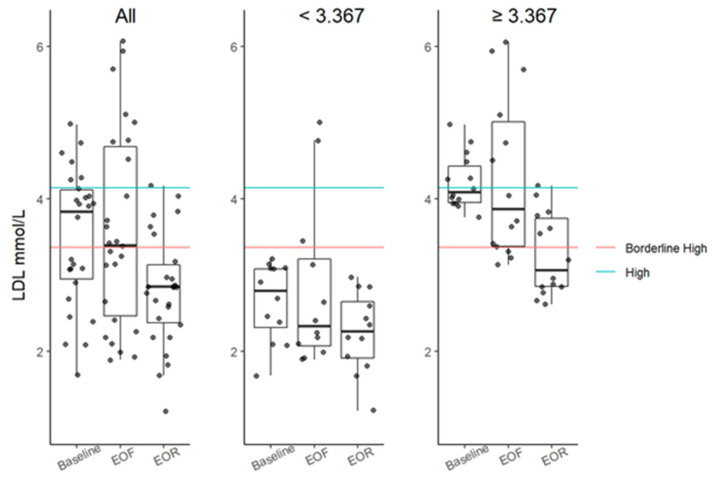
Serum LDL boxplots at baseline, EOF, and EOR. Data further categorized based on the high LDL threshold (≥3.367 mmol/L; red line). LDL between 3.367 and 4.1181 mmol/L is considered borderline high. LDL between 4.144 mmol/L (blue line) and 4.8951 mmol/L is considered high. Boxplots represent the minimum non-outlier value (lower whisker) whiskers, the first quartile (lowest point of the box), the median (horizontal line inside the box), the third quartile (highest point of the box), and the maximum non-outlier value (upper whisker). Data points that extend past the whiskers are outliers. EOF, end of fast; EOR, end of refeed; LDL, serum low-density lipoprotein; mmol, millimole; L, liter.

There was a clinically meaningful decrease in the median total cholesterol, from moderately elevated at baseline (5.67 mmol/L) to normal at EOR (4.87 mmol/L), which was significant with a MOD of −0.52 mmol/L (*p* = 0.0031; Table 1). There was no difference in the total cholesterol from baseline to EOF, with a MOD of 0 (*p* = 2.43; Table 1). There was also a significant decrease in low-density lipoprotein (LDL) from baseline to EOR with a MOD of −0.75 mmol/L (*p* = 0.0004; Table 1 and Figure 5). Participants with baseline LDL > 3.11 mmol/L (*n* = 16) had an even greater decrease in median LDL, with values of 4.03, 3.87, and 2.91 mmol/L at baseline, EOF, and EOR, respectively Table 1. In this subgroup, differences were significant from baseline to EOR with a MOD of −0.92 mmol/L (*p* = 0.0018; Table 1 and Figure 5). The fasting duration did not have a significant impact on the LDL (*p* = 0.2180; Supplemental Table S7).

There were also significant differences in high-density lipoprotein (HDL), very-low-density lipoprotein (VLDL), and triglycerides from baseline to EOR with MODs of −0.09 mmol/L (*p* = 0.0318), 0.30 mmol/L (*p* = 0.0023), and 0.62 mmol/L (*p* = 0.0011), respectively (Table 1). A 1-day increase in fasting duration was associated with a 0.02 mmol/L decrease in HDL (*p* = 0.0048), a 0.02 mmol/L increase in VLDL (*p* = 0.0023), and a 0.04 mmol/L increase in triglycerides (*p* = 0.0009; Supplemental Table S7), but these results were not found to be statistically significant in the sensitivity analyses (see Supplemental Tables S3 and S5).

### 3.5. High-Sensitivity C-Reactive Protein

Median hsCRP increased from 2.67 mg/L at baseline to 3.91 mg/L at EOF but had decreased to 1.68 mg/L at EOR with a MOD of −1.46 mg/L (*p* = 0.0041; Table 1). In participants with baseline hsCRP levels > 2 mg/L (*n* = 17), there was a significant difference between baseline and EOR and EOF and EOR, with MODs of −2.02 mg/L (*p* = 0.0068) and −2.43 mg/L (*p* = 0.0009), respectively (Table 1 and Supplemental Table S2). A 1-day increase in fasting duration and a 1-day increase in refeed duration were associated with, respectively, 0.22 mg/L (*p* = 0.0086) and 0.71 mg/L (*p* < 0.0001) decrease in hsCRP at EOR (Supplemental Table S7).

### 3.6. Homeostatic Model Assessment for Insulin Resistance (HOMA-IR)

The median glucose and insulin decreased from 4.94 and 40.2 pmol/L at baseline to 4.13 and 36 pmol/L at EOF and increased to 5.55 and 75.3 pmol/L at EOR, respectively (Table 1). Glucose levels changed significantly with MODs of −0.61 mmol/L (*p* = 0.0002) from baseline to EOF, 0.83 mmol/L (*p* = 0.0003) from baseline to EOR, and 1.42 mmol/L (*p* < 0.0001) from EOF to EOR (Table 1). Insulin increased significantly from baseline to EOR and from EOF to EOR with MODs of 41.7 pmol/L (*p* < 0.0001) and 36.9 pmol/L (*p* = 0.0001), respectively (Table 1).

Glucose and insulin values were used to calculate HOMA-IR (fasting insulin (µIU/L) × fasting glucose (nmol/L)/22.5). Accordingly, the median HOMA-IR decreased from 1.4 at baseline to 1.1 at EOF and then increased to 3.1 at EOR (*p* < 0.0001; Table 1), which were significant with MODs of −0.4 (*p* = 0.0329) from baseline to EOF, 2.0 (*p* < 0.0001) from baseline to EOR, and 1.8 (*p* < 0.0001) from EOF to EOR (Table 1). The fasting duration did not have a significant association with changes in glucose, insulin, or HOMA-IR from baseline to EOR (Supplemental Table S7).

## 4. Discussion

Prolonged fasting is a potential treatment for insulin resistance and cardiometabolic dysfunction as it reduces weight and improves other cardiometabolic markers without the use of pharmaceuticals or surgery [26,27,28,29]. Nonetheless, it is necessary to distinguish between temporary physiological adaptations to the total fasted state and sustained results that translate into health outcomes. By necessity, any sustained outcomes have to be studied in the context of the post-fast diet, which will also have an effect on cardiometabolic markers. Medically supervised, prolonged very-low-calorie or zero-calorie fasting methods typically require a controlled refeeding plan that varies depending on the method. However, there have been few human studies on the post-fast refeeding period and knowledge is limited. To this end, we conducted a single-arm, open-label, observational study to describe the effects of a minimum of 10 days of water-only fasting followed by a minimum of 5 days of refeeding with an exclusively whole-plant-food diet on select cardiometabolic markers.

Prolonged water-only fasting resulted in clinically meaningful and statistically significant reductions in weight, BMI, and AC that were sustained during refeeding. Our results support previous reports that water-only fasting reduces weight at a rate of 0.9 kg/day for approximately 1 week, decreasing to 0.3 kg/day by the third week due to combined loss of fat and lean mass [27]. Clinical observation and indirect measures of protein catabolism (e.g., urea excretion) indicate that muscle loss is minimal [30,31], suggesting that the lean mass loss is primarily from fluids. Decreased abdominal circumference is suggestive of a reduction in visceral fat, which is associated with insulin resistance and metabolic syndrome [32].

There was also a clinically meaningful and statistically significant reduction in SBP and DBP that is consistent with previous data showing that water-only fasting has a rapid antihypertensive effect that persists after refeeding [12,13]. Decreased blood pressure may be the result of the natriuresis observed during fasting [33,34,35], decreased sympathetic activation [36], and/or favorable changes in the sensitivity of the renin–angiotensin–aldosterone system (RAAS) [37,38]. No cases of electrolyte disturbance were reported, and participants were hydrated ad libitum during the study, making hypovolemia a possible but less likely explanation.

We did not observe significant changes in TC, HDL, LDL, VLDL, or TG after fasting. After refeeding, we observed a significant decrease in TC and LDL. LDL reduction was especially meaningful in participants with elevated baseline measures. The mechanism of action is beyond the scope of this study, but the reduction may be due to increased LDL receptor activity, potentially from cholesterol-deficient hepatocytes resulting from the fast [39,40] that did not occur until feeding resumed. This effect is notable for a one-time intervention and suggests the need for long-term follow-up studies to determine the duration of this effect, whether repeated cycles may have additive effects, and whether the effect is due to dietary intervention alone. We also observed an expected increase in VLDL and TG values after refeeding. Notably, a recent study reported similar findings to ours but found that TG levels decreased after post-fast refeeding. The refeeding diet differed in that it incorporated animal products and was higher in fat and lower in carbohydrates than the refeeding diet used in this study [41]. One possibility is that the high-carbohydrate refeeding diet used in this study contributed to the increased TG levels.

CRP is an acute-phase-reactant marker of inflammation and metabolic stress [42]. Prior reports have described elevated CRP during calorie-restricted fasting [29,43], which was attributed to circulating catecholamines. We did not observe significant changes in median hsCRP after fasting or refeeding, but longer fasting and refeed lengths were associated with decreased hsCRP. Participants with elevated hsCRP (>2 mg/L) at baseline had significantly lower hsCRP after refeeding and the refeed duration was positively correlated with decreased hsCRP. Whether the observed anti-inflammatory effects are due to diet alone or the combination of fasting and diet is unknown. Nonetheless, these findings suggest a hormetic effect on inflammation and require further investigation.

Insulin resistance is a common factor underlying the pathogenesis of cardiometabolic diseases that is difficult to directly measure. Therefore, we used HOMA-IR to assess for insulin resistance. HOMA-IR values above 2.5 are correlated with insulin resistance and cardiometabolic diseases [44]. Surprisingly, the median HOMA-IR, which was 1.4 at baseline and trending downward after fasting (1.1), had more than doubled after refeeding (3.1), suggesting that fasting may increase insulin resistance upon refeeding, at least in this overweight/obese non-diabetic population. Indeed, prolonged fasting may result in temporary physiological changes, such as downregulation of insulin receptor expression [45], elevated VLDL and TG levels [16,46], and metabolic inflammation in adipose tissue [47], which could temporarily affect insulin sensitivity during refeeding. We speculate that the observed insulin resistance is a temporary, rebound phenomenon caused by a reversal of the metabolic switch from ketosis back to glycolysis upon refeeding.

This study has several limitations, including that there were no “healthy” control populations, nor was there a dietary intervention arm. A metabolically healthy control population is necessary to determine if the observed effects, especially on post-fast glucose and insulin levels, are a transient response to fasting or specific to metabolic dysfunction. A dietary control population is necessary to assess if some of the changes (e.g., reduction of hsCRP) observed only at the end of refeed are due to diet alone. The data were also collected from a single center with a small sample size and, thus, external validity cannot be assessed. An additional limitation is the lack of long-term follow-up, which is currently being addressed in an ongoing study with an additional 6-week follow-up visit.

Ultimately, 46% (22/48) of the participants were unable to complete one or more study visits. Twelve of these participants had mild to moderate, common adverse events [10] that resulted in the premature suspension or termination of their fasting. It is noteworthy that in clinical practice, early termination or temporary suspension of a water-only fast with the intake of vegetable broth or juice happens as needed and is not necessarily an indicator that the water-only fast was intolerable or ineffective. Nonetheless, water-only fasting has been criticized for difficulty with patient tolerance [48], which has resulted in various modified fasting protocols that allow a minimal amount of caloric intake (<500 kcal per day) while preserving ketosis (e.g., fasting mimicking diet [28,49] and Buchinger fast [26]). Whether a clinically meaningful difference in health benefits exists between these protocols (water-only vs. calorie-restricted fasting) is unknown and should be pursued in randomized trials.

We chose a minimal fast length of ≥10 days because this length is considered minimally sufficient for the population based on anecdotal evidence from clinical practice. However, optimal fast duration has not been thoroughly investigated and likely varies based on individual demographics and treatment purpose. For example, fast duration was not correlated with the reduction in LDL but was strongly correlated with the reduction in abdominal circumference. Although a major strength of this study is that the prolonged water-only fasting and refeeding protocol was supervised in a domiciled setting, larger samples size and a broader range of fast lengths (i.e., 2–40 days) are necessary to address this question.

## 5. Conclusions

The increasing prevalence of cardiometabolic disease suggests the need for additional treatments. Prolonged water-only fasting may be an alternative, but there are many open questions about the long-term health effects and durability of outcomes. To this end, we measured markers of cardiometabolic health during the fasting and post-fast refeeding periods. Our results suggest that fasting combined with an exclusively whole-plant-food refeeding diet may be an effective nonpharmacological intervention for overweight/obese non-diabetic patients as we observed improvements in cardiometabolic risk factors, such as increased AC, elevated LDL cholesterol, and elevated CRP. However, we also found that refeeding on an exclusively whole-plant-food diet that does not limit carbohydrates may induce an insulin-resistant state. Long-term follow-up studies are necessary to assess if improvements are sustained and if fasting-associated insulin resistance is a transient rebound phenomenon.

## Figures and Tables

**Table 1:** The effects of fasting and refeeding on cardiometabolic markers.

	Median (IQR)			Friedman Test	Median of Differences (95% CI)		
					Bonferroni Corrected *p* Value		
	Baseline	EOF	EOR	Fr (*p* Value)	EOF Baseline	EOR Baseline	EOR–EOF
**Weight, kg**	87.2 (75.1–99.5)	78.3 (66.2–88.2)	79.5 (67.3–89.0)	43 (<0.0001)	−9.0 (−9.6, −8.0) <0.0001	−7.3 (−8.5, −6.1) <0.0001	1.3 (0.9, 2.0) 0.0006
**BMI, kg/m^2^** (18.5–24.9 kg/m^2^)	30.7 (27.5–35.3)	27.6 (24.6–31.5)	28.8 (25.1–31.9)	43 (<0.0001)	−3.2 (−3.4, −2.8) <0.0001	−2.5 (−3.1, −2.2) <0.0001	0.5 (0.3, 0.7) 0.0001
**AC, cm** (<101.6 cm for men and <88.9 cm for women)	97.6 (93.8–108.3)	90.7 (84.9–100.6)	90.8 (84.8–102.3)	46 (<0.0001)	−7.1 (−9.2, −6.4) <0.0001	−5.7 (−7.5, −4.9) <0.0001	1.7 (1.1, 2.6) 0.0054
**SBP, mmHg** (<130 mmHg)	130 (115–153)	119 (113–126)	117 (107–123)	13 (0.0017)	−14 (−26, −6) 0.0054	−13 (−27, −9) 0.0012	−3 (−6, 3) 1.5507
**DBP, mmHg** (<80 mmHg)	80 (76–87)	83 (71–85)	77 (71–82)	7 (0.0368)	−3 (−6, 2) 1.4533	−6 (−8, −1) 0.0874	−3 (−6, 1) 0.6872
**TC, mmol/L** (3.24–5.18 mmol/L)	5.67 (4.71–6.18)	5.27 (4.29–6.03)	4.87 (4.46–5.26)	7 (0.0280)	0 (−0.47, 0.35) 2.4280	−0.52 (−0.88, −0.23) 0.0031	−0.45 (−0.92, −0.08) 0.0667
**HDL, mmol/L** (≥1.17 mmol/L for men and ≥1.30 mmol/L for women)	1.28 (1.04–1.52)	1.14 (0.96–1.23)	1.09 (0.98–1.29)	9 (0.0094)	−0.12 (−1.26, −0.03) 0.0544	−0.09 (−0.22, −0.04) 0.03176	0.01 (−0.08, 0.12) 2.0988
**LDL, mmol/L** (<2.59 mmol/L)	3.83 (2.95–4.12)	3.39 (2.47–4.68)	2.85 (2.38–3.13)	19 (<0.0001)	−0.03 (−0.36, 0.48) 2.4666	−0.75 (−97, −0.45) 0.0004	−0.82 (−1.14, −0.41) 0.0010
**LDL > 3.11 mmol/L** ^‡^	4.03 (3.93–4.33)	3.87 (3.35–5.02)	2.91 (2.83–3.66)	16 (0.0004)	−0.13 (−0.63, 0.71) 2.1172	−0.92 (−1.20, −0.61) 0.0018	−0.96 (−1.53, −0.52) 0.0044
**VLDL, mmol/L** (<0.78 mmol/L)	0.63 (0.49–0.78)	0.66 (0.60–0.74)	0.87 (0.75–1.04)	20 (<0.0001)	0.03 (−0.10, 0.13) 1.7424	0.30 (0.13, 0.39) 0.0023	0.26 (0.17, 0.36) 0.0002
**TG, mmol/L** (<1.70 mmol/L)	1.39 (1.06–1.68)	1.44 (1.30–1.63)	1.91 (1.65–2.28)	21 (<0.0001)	0.04 (−0.21, 0.27) 1.7813	0.62 (0.30, 0.84) 0.0011	0.55 (0.35, 0.77) <0.0001
**hsCRP, mg/L** (<1.0 mg/L)	2.67 (1.00–4.60)	3.91 (1.84–7.09)	1.68 (0.79–3.44)	9 (0.0088)	0.60 (−0.07, 1.69) 0.2526	−0.55 (−2.65, −0.07) 0.0897	−1.46 (−3.35, 0.87) 0.0041
**hsCRP > 2 mg/L** ^‡ ‡^	3.64 (2.70–7.01)	5.19 (3.68–8.06)	2.54 (1.56–3.47)	15 (0.0007)	1.07 (−2.05, 2.81) 1.3757	−2.02 (−4.71, −0.81) 0.0068	−2.43 (−5.01, 1.53) 0.0009
**Glucose, mmol/L** (<7.8 mmol/L)	4.94 (4.56–5.05)	4.13 (3.90–4.66)	5.55 (5.05–5.97)	38 (<0.0001)	−0.61 (−0.94, −0.44) 0.0002	0.83 (0.47, 1.05) 0.0003	1.42 (1.08, 1.83) < 0.0001
**Insulin, pmol/L** (<102 pmol/L)	40.2 (30.8–64.5)	36 (23.4–44.3)	75.3 (62.0–126.8)	34 (<0.0001)	−7.2 (−24.0, 1.2) 0.1912	41.7 (32.7, 60.9) <0.0001	36.9 (32.4, 86.7) 0.0001
**HOMA-IR** (<1 is optimal)	1.4 (1.0–2.6)	1.1 (0.6–1.4)	3.1 (2.2–6.1)	37 (<0.0001)	−0.4 (−1.2, −0.1) 0.0329	2.0 (1.5, 2.9) <0.0001	1.8 (1.5, 4.0) 0.0001

*N* = 26. Data are represented as median (range). Reference ranges for normal values are given below the respective parameters (1–4). The 95% CI and *p*-values for the median of differences are from Wilcoxon signed-rank analysis. IQR, interquartile range; CI, confidence interval; EOF, end of fast; EOR, end of refeed; Fr, Friedman chi-squared with 2 degrees of freedom; kg, kilogram; m, meter; cm, centimeter; SBP, systolic blood pressure; DBP, diastolic blood pressure; mmHg, millimeters of mercury; mg, milligram; L, liter; mmol, millimole; pmole, picomole; TC, total cholesterol; HDL, high-density lipoprotein; LDL, low-density lipoprotein; VLDL, very-low-density lipoprotein; TG, triglycerides; hsCRP, high-sensitivity C-reactive protein; HOMA-IR, homeostatic model assessment for insulin resistance. ‡ *N* = 16. ‡ ‡ *N* = 17.

## Data Availability

The data that support the findings of this study are available from the corresponding author, E.S., upon reasonable request.

## Supplementary Materials

The following supporting information can be downloaded at: https://www.mdpi.com/article/10.3390/nu14061183/s1. Table S1 Baseline characteristics comparing subjects who completed protocol versus those who did not complete protocol; Table S2 Repeated Measures ANOVA, a sensitivity analysis for the Friedman test; Table S3 Change Score Regression (no outliers), a sensitivity analysis for Siegel repeated medians; Table S4 Change Score Regression (with outliers), a sensitivity analysis for Siegel repeated medians; Table S5 Baseline Adjusted Regression (no outliers), a sensitivity analysis for Siegel repeated medians; Table S6 Baseline adjusted regression (with outliers), a sensitivity analysis for Siegel repeated medians; Table S7 Linear regression (with outliers), a sensitivity analysis for linear regression (no outliers).
