# Supplementary material for: The Effects of Prolonged Water-Only Fasting and Refeeding on Markers of Cardiometabolic Risk
Source: Nutrients. 2022 Mar 11;14(6):1183. doi: 10.3390/nu14061183 (PMC8951503; doi:10.3390/nu14061183)
Supplement: Supplementary file 1 [file nutrients-14-01183-s001.zip › nutrients-1570396-supplementary.pdf]

Table S1. Baseline characteristics comparing subjects who completed protocol versus those who did not complete protocol.

	Completed Study		Did not complete study	
Biological sex	Male	Female	Male	Female
N=	6	20	6	16
Age, years, mean (SD)	57(10)	58(8)	51(7)	58(9)
BMI, kg/m², mean (SD)	30 (3)	30 (6)	31(4)	33(7)
Abdominal circumference, mean, cm (SD)	104(8)	93(12)	109(10)	99(15)
Systolic Blood pressure, mean, mmHg (SD)	128(16)	124(19)	125(10)	126(21)
Diastolic Blood pressure, mean, mmHg (SD)	84 (8)	79 (8)	83 (6)	77 (6)
Past Medical History				
Asthma	0	2	0	1
Anxiety	0	4	3	1
Coronary Artery Disease	0	0	1	1
Impaired Glucose	2	1	0	1
Hyperlipidemia	4	8	5	4
Hypertension	2	7	2	3
Migraine	0	1	0	0
Obesity	3	12	5	5
Sleep Apnea	0	1	0	1
Osteoarthritis	1	3	0	1

Table S2. Repeated Measures ANOVA, a sensitivity analysis for the Friedman test.

	**Mean (SD)**			**RMA**	**Mean Difference (Pooled SD)** *Bonferroni Corrected P value*		
	**Baseline**	**EOF**	**EOR**	***P* value**	**EOF – Baseline**	**EOR – Baseline**	**EOR - EOF**
**Weight, kg**	88.9 (17.1)	80.1 (16.4)	81.7 (16.5)	<0.0001	-8.8 (16.8)<br><0.0001	-7.3 (16.8)<br><0.0001	1.6 (16.5)<br>0.004
**BMI, kg/m2**	31.9 (5.4)	28.8 (5.2)	29.3 (5.1)	<0.0001	-3.2 (5.3)<br><0.0001	-2.6 (5.2)<br><0.0001	0.5 (5.1)<br>0.0017
**Abdominal circumference, cm**	99.4 (11.2)	91.3 (11.4)	92.9 (10.9)	<0.0001	-8.1 (11.3)<br><0.0001	-6.5 (11.1)<br><0.0001	1.6 (11.2)<br>0.076 $^{\lambda}$
**Systolic BP, mmHg**	134.6 (22.9)	117.9 (9.9)	116.8 (12.6)	0.0003	-16.7 (17.6)<br>0.0026	-17.8 (18.5)<br>0.0017	-1.2 (11.3)<br>1

**Diastolic BP, mmHg**	81.2 (8.8)	79.5 (8.5)	77.5 (7.9)	0.1280 $^{\lambda}$	-	-	-
**Total cholesterol, mg/dl**	210.0 (39.1)	208.2 (50.1)	187.6 (29.4)	0.0030	-1.8 (44.9)<br>1	-22.4 (34.6)<br>0.0011	-20.5 (41.1)<br>0.0288$^{\lambda}$
**HDL, mg/dL**	49.3 (12.6)	43.6 (9.3)	44.2 (8.8)	0.0040	-5.7 (11.1)<br>0.0300$^{\lambda}$	-5.1 (10.9)<br>0.0130	0.6 (9.0)<br>1
**LDL, mg/dL**	135.0 (34.8)	139.0 (49.6)	108.2 (28.5)	<0.0001	4.0 (42.8)<br>1	-26.9 (31.8)<br><0.0001	-30.8 (40.5)<br>0.0004
**LDL > 120mg/dL ‡**	157.8 (18.9)	162.9 (42.0)	123.2 (21.4)	0.0008	5.1 (32.5)<br>1	-34.6 (20.2)<br><0.0001	-39.8 (33.3)<br>0.0013
**VLDL, mg/dL**	25.7 (10.3)	25.5 (4.5)	35.3 (8.6)	<0.0001	-0.1 (8.0)<br>1	9.6 (9.5)<br>0.0011	9.7 (6.9)<br><0.0001
**Triglycerides, mg/dL**	128.2 (51.5)	127.3 (22.6)	176.8 (43.1)	<0.0001	-0.8 (39.7)<br>1	48.7 (47.5)<br>0.0010	49.5 (34.4)<br><0.0001
**hsCRP, mg/L**	3.97 (4.36)	4.56 (3.41)	2.38 (1.87)	0.0040	0.59 (3.91)<br>1	-1.59 (3.36)<br>0.0793	-2.18 (2.75)<br>0.0028
**hsCRP > 2mg/dL‡‡**	5.69 (4.53)	6.15 (3.07)	2.81 (1.80)	0.0010	0.45 (3.87)<br>1	-2.89 (3.45)<br>0.0103	-3.34 (2.51)<br>0.0008
**Glucose, nmol/L**	4.95 (0.57)	4.21 (0.62)	5.66 (0.93)	<0.0001	-0.74 (0.60)<br><0.0001	0.71 (0.77)<br><0.0001	1.45 (0.79)<br><0.0001
**Insulin**	8.1 (4.3)	6.3 (4.1)	17.1 (11.4)	<0.0001	-1.8 (4.2)<br>0.3031	9.0 (8.6)<br><0.0001	10.8 (8.5)<br>0.0002
**HOMA-IR**	0.43 (0.58)	-0.03 (0.76)	1.28 (0.65)	<0.0001	-0.47 (0.68)<br>0.0095	0.85 (0.61)<br><0.0001	1.32 (0.71)<br><0.0001

N = 26. Omnibus p-values are from RMA; post-hoc p-values are from paired t-tests with a Bonferroni correction. SD, standard deviation; EOF, end-of-fast; EOR, end-of-refeed; kg, kilogram; m, meter; cm, centimeter; BP, blood pressure; mmHg, millimeter mercury; mg, milligram; dL, deciliter; HDL, high-density lipoprotein; LDL, low-density lipoprotein; VLDL, very-low-density lipoprotein; hsCRP, high-sensitivity C-reactive protein; HOMA-IR, homeostatic model assessment for insulin resistance; HOMA-IR was log transformed prior to modeling. ‡ *N* = 16. ‡ ‡ *N* = 17. ζ = Direction of a statistically significant coefficient diverges from a statistically significant coefficient from the main model of interest. λ = Statistical significance of p-value diverges from the main model of interest.

Table S3. Change Score Regression (no outliers), a sensitivity analysis for Siegel repeated medians

Model		Sample Size	R^2	Coefficients		t value	Pr(>|t|)
**Change Score**	**Dependent Variable**			**β**	**S.E.**		
Abdominal Circumference, cm[‡]	Length of Fast, days	25	0.52	-0.41	0.08	-4.95	<0.0001
Abdominal Circumference, cm	Length of Refeed, days	25	0.19	-0.31	0.13	-2.35	0.0276
Abdominal Circumference, cm	Length of Total, days	25	0.51	-0.27	0.06	-4.93	<0.0001
LDL, mg/dL	Length of Fast, days	26	0.01	-0.51	0.94	-0.55	0.5902
LDL, mg/dL	Length of Refeed, days[^]	26	0.01	0.49	1.16	0.42	0.68
LDL, mg/dL	Length of Total, days	26	0.00	-0.09	0.63	-0.14	0.894
hsCRP, mg/L[‡]	Length of Fast, days	25	0.18	-0.22	0.10	-2.24	0.0352
hsCRP, mg/L[‡]	Length of Refeed, days[^]	25	0.10	-0.21	0.13	-1.61	0.1204
hsCRP, mg/L	Length of Total, days	25	0.21	-0.16	0.07	-2.45	0.0224
Weight, kg	Length of Fast, days	26	0.29	-0.32	0.10	-3.14	0.0045
Weight, kg	Length of Refeed, days	25	0.37	-0.37	0.10	-3.65	0.0013
Weight, kg	Length of Total, days	25	0.52	-0.24	0.05	-4.98	<0.0001
BMI, kg/m2[‡]	Length of Fast, days	25	0.52	-0.13	0.03	-5.01	<0.0001
BMI, kg/m2	Length of Refeed, days	25	0.39	-0.14	0.04	-3.86	0.0008
BMI, kg/m2	Length of Total, days	25	0.68	-0.10	0.01	-6.97	<0.0001
Systolic BP, mmHg	Length of Fast, days	26	0.02	0.59	0.89	0.66	0.5132
Systolic BP, mmHg	Length of Refeed, days	26	0.00	-0.12	1.11	-0.11	0.9161
Systolic BP, mmHg	Length of Total, days	26	0.01	0.23	0.60	0.38	0.705
Diastolic BP, mmHg[‡]	Length of Fast, days[^]	25	0.11	0.51	0.31	1.65	0.1124
Diastolic BP, mmHg	Length of Refeed, days	26	0.02	-0.31	0.49	-0.63	0.533
Diastolic BP, mmHg	Length of Total, days	26	0.09	0.08	0.26	0.31	0.761
Total Cholesterol, mg/dL	ΔLDL, mg/dL	26	0.74	0.98	0.12	8.29	<0.0001
Total Cholesterol, mg/dL	ΔHDL, mg/dL[^]	26	0.07	0.87	0.66	1.33	0.1973
Total Cholesterol, mg/dL	ΔVLDL, mg/dL[^]	26	0.15	0.89	0.44	2.04	0.0525
Total Cholesterol, mg/dL	ΔTriglycerides, mg/dL	26	0.15	0.18	0.09	2.08	0.0481
Total Cholesterol, mg/dL	Length of Fast, days	26	0.00	-0.13	1.08	-0.12	0.906
Total Cholesterol, mg/dL	Length of Refeed, days[^]	26	0.02	0.92	1.32	0.70	0.4905
Total Cholesterol, mg/dL	Length of Total, days	26	0.00	0.21	0.72	0.30	0.77
Triglycerides, mg/dL	Length of Fast, days[^]	26	0.10	3.63	2.21	1.64	0.1133
Triglycerides, mg/dL	Length of Refeed, days[^]	26	0.06	3.40	2.79	1.22	0.235
Triglycerides, mg/dL	Length of Total, days[^]	26	0.12	2.62	1.46	1.79	0.0861
HDL, mg/dL	Length of Fast, days[^]	26	0.04	-0.32	0.32	-1.01	0.3215
HDL, mg/dL	Length of Refeed, days	26	0.01	-0.22	0.40	-0.55	0.591
HDL, mg/dL	Length of Total, days[^]	26	0.04	-0.21	0.21	-0.97	0.34
VLDL, mg/dL	Length of Fast, days[^]	26	0.02	0.02	0.03	0.62	0.54
VLDL, mg/dL	Length of Refeed, days[^]	26	0.05	0.65	0.56	1.16	0.257
VLDL, mg/dL	Length of Total, days[^]	26	0.11	0.50	0.29	1.72	0.0976
Glucose,nmol/L	Length of Fast, days	26	0.02	0.28	0.45	0.62	0.54
Glucose, nmol/L	Length of Refeed, days	26	0.06	-0.04	0.03	-1.26	0.2212
Glucose, nmol/L	Length of Total, days	26	0.66	0.00	0.02	-0.25	0.807
Insulin[‡]	Length of Fast, days	25	0.00	-0.02	0.25	-0.09	0.9288

Insulin	Length of Refeed, days	25	0.02	0.18	0.27	0.65	0.523
Insulin	Length of Total, days	25	0	0.05	0.16	0.33	0.747
HOMA-IR	Length of Fast, days	26	0.06	0.02	0.02	1.28	0.214
HOMA-IR	Length of Refeeding, days$^{\lambda}$	26	0.01	-0.01	0.02	-0.39	0.703
HOMA-IR	Length of Total, days	26	0.44	0.01	0.01	0.62	0.54
HOMA-IR$^{\alpha}$	Length of Fast, days	26	0.03	0.02	0.03	0.79	0.44
HOMA-IR$^{\alpha}$	Length of Refeeding, days	26	0.01	-0.02	0.04	-0.47	0.6453
HOMA-IR$^{\alpha}$	Length of Total, days	26	0	0.01	0.02	0.27	0.791

N = 26. ‡ = 25 observations (i.e., *N* = 25). Change scores are from baseline to EOR. α = Change score from baseline to EOF. λ = Statistical significance of p-value diverges from the main model of interest.

Table S4. Change Score Regression (with outliers), a sensitivity analysis for Siegel repeated medians

Model		R^2	Coefficients		t value	Pr(>|t|)
**Change Score**	**Baseline**		**β**	**S.E.**		
Abdominal Circumference, cm	Length of Fast, days	0.36	-0.45	0.12	-3.65	0.0013
Abdominal Circumference, cm	Length of Refeed, days$^{\lambda}$	0.10	-0.29	0.18	-1.62	0.1179
Abdominal Circumference, cm	Length of Total, days	0.32	-0.29	0.09	-3.39	0.0024
hsCRP, mg/L	Length of Fast, days$^{\lambda}$	0.14	-0.24	0.12	-1.94	0.0647
hsCRP, mg/L	Length of Refeeding, days$^{\lambda}$	0.06	-0.19	0.16	-1.2	0.2404
hsCRP, mg/L	Length of Total, days$^{\lambda}$	0.14	-0.16	0.08	-1.98	0.0593
Weight, kg	Length of Refeed, days	0.29	-0.40	0.13	-3.14	0.0045
Weight, kg	Length of Total, days	0.43	-0.26	0.06	-4.21	0.0003
BMI, kg/m2	Length of Fast, days	0.43	-0.14	0.03	-4.28	0.0003
BMI, kg/m2	Length of Refeed, days	0.32	-0.15	0.05	-3.34	0.0027
BMI, kg/m2	Length of Total, days	0.55	-0.11	0.02	-5.47	<0.0001
Diastolic BP, mmHg	Length of Fast, days$^{\lambda}$	0.04	0.38	0.39	0.99	0.3329
Insulin	Length of Fast, days$^{\lambda}$	0.16	0.70	0.32	2.17	0.0404
Insulin	Length of Refeed, days	0.06	0.53	0.42	1.27	0.218
Insulin	Length of Total, days$^{\lambda}$	0.16	0.47	0.21	2.17	0.0398

N = 26. Change scores are from baseline to EOR. λ = Statistical significance of p-value diverges from the main model of interest.

Table S5. Baseline Adjusted Regression (no outliers), a sensitivity analysis for Siegel repeated medians.

Model		Sample Size	R^2	Coefficients		t value	Pr(>|t|)
**Dependent Variable**	**Baseline**			**β**	**S.E.**		
Abdominal Circumference, cm‡	Abdominal Circumference, cm	25	0.97	0.94	0.04	25.07	<0.0001
	Length of Fast, days			-0.40	0.08	-5.03	<0.0001

Abdominal Circumference, cm	Abdominal Circumference, cm	25	0.94	0.96	0.05	18.22	<0.0001
	Length of Refeed, days^λ			-0.28	0.14	-2.01	0.0574
Abdominal Circumference, cm	Abdominal Circumference, cm	25	0.96	0.96	0.04	24.27	<0.0001
	Length of Total, days			-0.26	0.06	-4.64	0.0001
LDL, mg/dL	LDL, mg/dL	26	0.52	0.59	0.12	4.87	<0.0001
	Length of Fast, days			0.07	0.80	0.09	0.933
LDL, mg/dL	LDL, mg/dL	26	0.55	0.56	0.12	4.84	<0.0001
LDL, mg/dL	LDL, mg/dL	26	0.53	0.57	0.12	4.73	<0.0001
	Length of Total, days			0.38	0.53	0.71	0.485
hsCRP, mg/L‡	hsCRP, mg/L	25	0.33	0.32	0.10	3.19	0.0042
	Length of Fast, days ^λ			-0.04	0.06	-0.64	0.5306
hsCRP, mg/L‡	hsCRP, mg/L	25	0.33	0.31	0.09	3.23	0.0038
	Length of Refeeding, days ^λ			-0.04	0.07	-0.56	0.5812
hsCRP, mg/L	hsCRP, mg/L	25	0.33	0.32	0.10	3.23	0.0038
	Length of Total, days^λ			-0.03	0.04	-0.76	0.4573
Weight, kg	Weight, kg	26	0.98	0.92	0.03	31.92	<0.0001
	Length of Fast, days			-0.38	0.09	-4.04	0.0005
Weight, kg	Weight, kg	25	0.99	0.95	0.03	37.93	<0.0001
	Length of Refeed, days			-0.32	0.10	-3.18	0.0044
Weight, kg	Weight, kg	25	0.99	0.93	0.02	53.37	<0.0001
	Length of Total, days			-0.24	0.04	-6.28	<0.0001
BMI, kg/m2	BMI, kg/m2	25	0.99	0.93	0.02	43.98	<0.0001
	Length of Fast, days			-0.13	0.02	-6.01	<0.0001
BMI, kg/m2	BMI, kg/m2	25	0.98	0.96	0.03	31.58	<0.0001
	Length of Refeed, days			-0.12	0.03	-3.19	0.0043
BMI, kg/m2	BMI, kg/m2	25	0.99	0.95	0.02	48.74	<0.0001
	Length of Total, days			-0.09	0.01	-6.97	<0.0001
Systolic BP, mmHg	Systolic BP, mmHg	26	0.07	0.14	0.11	1.24	0.229

	Length of Fast, days			-0.11	0.49	-0.22	0.831
Systolic BP, mmHg	Systolic BP, mmHg	26	0.07	0.14	0.11	1.29	0.211
	Length of Refeed, days			0.12	0.60	0.20	0.85
Systolic BP, mmHg	Systolic BP, mmHg	26	0.07	0.14	0.11	1.29	0.211
	Length of Total, days			-0.01	0.32	-0.04	0.972
Diastolic BP, mmHg	Diastolic BP, mmHg	25	0.21	0.39	0.16	2.42	0.024
	Length of Fast, days [λ]			0.15	0.26	0.58	0.568
Diastolic BP, mmHg	Diastolic BP, mmHg	26	0.12	0.22	0.18	1.23	0.2316
	Length of Refeed, days			-0.44	0.37	-1.19	0.2459
Diastolic BP, mmHg	Diastolic BP, mmHg	26	0.09	0.20	0.19	1.07	0.295
	Length of Total, days			-0.15	0.21	-0.72	0.4776
Total Cholesterol, mg/dL	Total Cholesterol, mg/dL	26	0.82	0.80	0.08	10.14	<0.0001
	ΔLDL, mg/dL			0.81	0.13	6.35	<0.0001
Total Cholesterol, mg/dL	Total Cholesterol, mg/dL	26	0.53	0.55	0.11	5.04	<0.0001
	ΔHDL, mg/dL[λ]			0.57	0.51	1.11	0.2806
Total Cholesterol, mg/dL	Total Cholesterol, mg/dL	26	0.53	0.58	0.11	5.04	<0.0001
	ΔVLDL, mg/dL[λ]			0.43	0.37	1.14	0.27
Total Cholesterol, mg/dL	Total Cholesterol, mg/dL	26	0.51	0.52	0.11	4.62	0.0001
	Length of Fast, days			0.58	0.83	0.70	0.4909
Total Cholesterol, mg/dL	Total Cholesterol, mg/dL	26	0.55	0.51	0.11	4.81	<0.0001
	Length of Refeed, days[λ]			1.49	0.98	1.53	0.1404
Total Cholesterol, mg/dL	Total Cholesterol, mg/dL	26	0.54	0.50	0.11	4.62	0.0001

	Length of Total, days			0.71	0.54	1.31	0.2043
Total Cholesterol, mg/dL	Total Cholesterol, mg/dL	26	0.53	0.58	0.11	5.07	<0.0001
	ΔTriglycerides, mg/dL[λ]			0.09	0.07	1.20	0.2422
Triglycerides, mg/dL	Triglycerides, mg/dL	26	0.15	0.21	0.16	1.30	0.2075
	Length of Fast, days [λ]			2.63	1.60	1.65	0.1134
Triglycerides, mg/dL	Triglycerides, mg/dL	26	0.09	0.20	0.17	1.19	0.2474
	Length of Refeed, days[λ]			2.10	2.04	1.03	0.3153
Triglycerides, mg/dL	Triglycerides, mg/dL	26	0.15	0.22	0.16	1.35	0.19
	Length of Total, days[λ]			1.80	1.07	1.69	0.1055
HDL, mg/dL	HDL, mg/dL	26	0.58	0.53	0.09	5.66	<0.0001
	Length of Fast, days[λ]			-0.18	0.23	-0.80	0.4337
HDL, mg/dL	HDL, mg/dL	26	0.58	0.52	0.09	5.60	<0.0001
	Length of Refeed, days			-0.24	0.28	-0.86	0.3992
HDL, mg/dL	HDL, mg/dL	26	0.59	0.53	0.09	5.71	<0.0001
	Length of Total, days[λ]			-0.15	0.15	-1.00	0.3264
VLDL, mg/dL	VLDL, mg/dL	26	0.14	0.22	0.16	1.33	0.1976
	Length of Fast, days[λ]			0.52	0.32	1.61	0.1212
VLDL, mg/dL	VLDL, mg/dL	26	0.09	0.20	0.17	1.22	0.2363
	Length of Refeed, days[λ]			0.40	0.41	0.97	0.3406
VLDL, mg/dL	VLDL, mg/dL	26	0.15	0.22	0.16	1.37	0.1831
	Length of Total, days[λ]			0.35	0.21	1.63	0.1173
Glucose, nmol/L	Glucose, nmol/L	26	0.53	1.19	0.23	5.08	<0.0001
	Length of Fast, days			0.02	0.03	0.60	0.556
Glucose, nmol/L	Glucose, nmol/L	25	0.56	1.18	0.23	5.18	<0.0001
	Length of Refeed, days			-0.04	0.03	-1.22	0.234
Glucose, nmol/L	Glucose, nmol/L	26	0.53	1.19	0.24	5.06	<0.0001
	Length of Total, day			0.00	0.02	-0.25	0.808
Insulin	Insulin	25	0.56	1.39	0.26	5.26	<0.0001

	Length of Fast, days			0.01	0.25	0.04	0.965
Insulin	Insulin	26	0.56	1.38	0.28	4.89	<0.0001
	Length of Refeed, days			0.04	0.28	0.15	0.88
Insulin	Insulin	25	0.56	1.39	0.27	5.20	<0.0001
	Length of Total, days			0.02	0.16	0.12	0.909
HOMA-IR	HOMA-IR	26	0.61	0.83	0.15	5.65	<0.0001
	Length of Fast, days			0.02	0.02	1.39	0.179
HOMA-IR	HOMA-IR	26	0.57	0.85	0.16	5.32	<0.0001
	Length of Refeeding, days$^{\lambda}$			0.00	0.02	-0.08	0.939
HOMA-IR	HOMA-IR	26	0.59	0.82	0.15	5.31	<0.0001
	Length of Total, days			0.01	0.01	0.88	0.3864
HOMA-IR$^{\alpha}$	HOMA-IR	26	0.61	0.83	0.15	5.65	<0.0001
	Length of Refeeding, days			0.02	0.02	1.39	0.179
HOMA-IR$^{\alpha}$	HOMA-IR	26	0.57	0.85	0.16	5.32	<0.0001
	Length of Refeeding, days			0.00	0.02	-0.08	0.939
HOMA-IR$^{\alpha}$	HOMA-IR	26	0.59	0.82	0.15	5.31	<0.0001
	Length of Total, days			0.01	0.01	0.88	0.3864

N = 26. ‡ = 25 observations (i.e., N = 25). Dependent variable measured at EOR. ζ = Direction of a statistically significant coefficient diverges from a statistically significant coefficient from the main model of interest. λ = Statistical significance of p-value diverges from the main model of interest.

Table S6. Baseline adjusted regression (with outliers), a sensitivity analysis for Siegel repeated medians

Model		R^2	Coefficients		t value	Pr(>$\lvert t \rvert$)
**Change Score**	**Baseline**		**β**	**S.E.**		
Abdominal Circumference, cm	Abdominal Circumference, cm	0.92	0.92	0.06	16.16	<0.0001
	Length of Fast, days		-0.44	0.12	-3.61	0.0015
Abdominal Circumference, cm	Abdominal Circumference, cm	0.88	0.94	0.07	13.05	<0.0001
	Length of Refeed, days$^{\lambda}$		-0.25	0.19	-1.31	0.204
Abdominal Circumference, cm	Abdominal Circumference, cm	0.91	0.95	0.06	15.56	<0.0001
	Length of Total, days		-0.27	0.09	-3.14	0.0046
hsCRP, mg/L	hsCRP, mg/L	0.44	0.30	0.07	4.11	0.0004

	Length of Fast, days λ		-0.04	0.06	-0.59	0.564
hsCRP, mg/L	hsCRP, mg/L	0.43	0.29	0.07	4.18	0.0004
	Length of Refeeding, days λ		-0.04	0.07	-0.52	0.6094
hsCRP, mg/L	hsCRP, mg/L	0.44	0.30	0.07	4.16	0.0004
	Length of Total, daysλ		-0.03	0.04	-0.69	0.4997
Weight, kg	Weight, kg	0.98	0.97	0.03	29.49	<0.0001
	Length of Refeed, days		-0.37	0.13	-2.79	0.0105
Weight, kg	Weight, kg	0.98	0.95	0.03	35.19	<0.0001
	Length of Refeed, days		-0.26	0.06	-4.47	0.0002
BMI, kg/m2	BMI, kg/m2	0.98	0.92	0.03	31.88	<0.0001
	Length of Fast, days		-0.14	0.03	-4.75	<0.0001
BMI, kg/m2	BMI, kg/m2	0.97	0.96	0.04	25.12	<0.0001
	Length of Refeed, days		-0.13	0.05	-2.71	0.0126
BMI, kg/m2	BMI, kg/m2	0.98	0.95	0.03	33.62	<0.0001
	Length of Total, days		-0.10	0.02	-5.20	<0.0001
Diastolic BP, mmHg	Diastolic BP, mmHg	0.07	0.23	0.19	1.19	0.2476
	Length of Fast, days λ		-0.03	0.32	-0.10	0.918
Insulin	Insulin	0.55	1.73	0.37	4.70	<0.0001
	Length of Fast, days λ		0.67	0.3	2.20	0.0386
Insulin	Insulin	0.46	1.67	0.44	3.84	0.0008
	Length of Refeed, days		0.27	0.44	0.61	0.5455
Insulin	Insulin	0.52	1.60	0.39	4.10	0.0004
	Length of Total, days		0.39	0.22	1.80	0.0849

N = 26. Dependent variable measured at EOR. λ = Statistical significance of p-value diverges from the main model of interest.

Table S7. Linear regression (with outliers), a sensitivity analysis for linear regression (no outliers)

Model		R^2	Coefficients		t value	Pr(>|t|)
End of Refeed	Baseline		β	S.E.		
ln HOMA-IR	ln HOMA-IR	0.57	0.85	0.15	5.69	<0.0001
ln HOMA-IR	Length of Fast, days	0.06	0.03	0.02	1.26	0.2210

ln HOMA-IR	Length of Refeeding, days	0.05	0.03	0.03	1.13	0.2685

N = 26. Results include outlier ID 24.
